# Using Tree-Based Machine Learning for Health Studies: Literature Review and Case Series

**DOI:** 10.3390/ijerph192316080

**Published:** 2022-12-01

**Authors:** Liangyuan Hu, Lihua Li

**Affiliations:** 1 Department of Biostatistics and Epidemiology, Rutgers University, Piscataway, NJ 08854, USA; 2 Department of Population Health Science and Policy, Icahn School of Medicine at Mount Sinai, New York, NY 10029, USA

**Keywords:** causal inference, variable selection, missing data, sensitivity analysis, ensemble methods

## Abstract

Tree-based machine learning methods have gained traction in the statistical and data science fields. They have been shown to provide better solutions to various research questions than traditional analysis approaches. To encourage the uptake of tree-based methods in health research, we review the methodological fundamentals of three key tree-based machine learning methods: random forests, extreme gradient boosting and Bayesian additive regression trees. We further conduct a series of case studies to illustrate how these methods can be properly used to solve important health research problems in four domains: variable selection, estimation of causal effects, propensity score weighting and missing data. We exposit that the central idea of using ensemble tree methods for these research questions is accurate prediction via flexible modeling. We applied ensemble trees methods to select important predictors for the presence of postoperative respiratory complication among early stage lung cancer patients with resectable tumors. We then demonstrated how to use these methods to estimate the causal effects of popular surgical approaches on postoperative respiratory complications among lung cancer patients. Using the same data, we further implemented the methods to accurately estimate the inverse probability weights for a propensity score analysis of the comparative effectiveness of the surgical approaches. Finally, we demonstrated how random forests can be used to impute missing data using the Study of Women’s Health Across the Nation data set. To conclude, the tree-based methods are a flexible tool and should be properly used for health investigations.

## 1. Introduction

Tree-based machine learning methods have gained wide popularity in the statistical and data science fields. There have been burgeoning applications showing that tree-based methods can generate better results than traditional methods. To name a few, biomarker discovery in proteomic studies [1], estimation of causal effects [2,3], prediction of healthcare cost [4], identification of key risk factors [5,6], and hospital performance evaluation [7]. In health studies, tree-based methods have not gained the same traction as in data science. To encourage the uptake of these methods in health research, we provide a primer on how tree-based methods can be properly used to solve four important and general statistical problems:

(i) variable selection;
(ii) estimation of causal effects;
(iii) propensity score weighting;
(iv) missing data.

We select these four general application areas because they share a common theme: they all require modeling the unknown and arbitrarily complex relationships among the response, treatment/exposure, and other covariates, for which the flexibility of tree-based models can be leveraged to improve the relevant results.

The variable selection problem (i) arises when one wishes to model the relationship between a response variable and a subset of candidate predictors, but there is uncertainty about which subset to use. The variable selection procedure typically involves selecting a subset of predictors that has the most impact on the fit of the model linking a response variable and a set of candidate predictors [8]. Variable selection is useful for discovering important risk factors that were previously less known for diseases of interest, identifying key confounding variables for a causal comparative effectiveness analysis, and reducing dimension of large genomic data sets. Turning to problem (ii), a popular way to estimate the causal treatment effect is via outcome modeling, which requires the prediction of the counterfactual outcomes from a model linking the observed outcomes and covariates [2,9,10,]. An additional utility of using tree-based methods for this problem is the exploration and estimation of treatment effect heterogeneity using the conditional models built for estimating the average causal effects [11,12]. For problem (iii), propensity score weighting, a selection model is needed to establish the relationship between the treatment assignment and confounding variables, and the propensity scores are then estimated from the fitted selection model [13,14]. Propensity score weighting is a technique widely used in controlling for selection biases in non-experimental studies. The treatment selection bias due to measured confounding can be effectively removed in the propensity score weighted pseudo-populations. Missing data (iv) are a pervasive problem in many health studies. There are three general missing data mechanisms: missing completely at random, missing at random, and missing not at random [15]. The missing completely at random assumption is unlikely in public health investigations. To deal with missing at random data, researchers often resort to imputation [16], which imputes a variable’s missing values from a model that regresses that variable against all other variables [17]. In the situation where the missingness depends on the missing data, the data are missing not at random. Sensitivity analysis [18] is a recommended approach to handle missing not at random by assessing the impact of assumptions about the missing data on inference. In this article, we exposit the utilization of tree-based methods for missing at random data. Imputation as a statistical technique for handing missing data has gained wide popularity for its generality. Thus, at the core of each statistical problem is modeling the unknown relationships between the response and the explanatory variables.

To demonstrate how tree-based machine learning techniques can be applied to address each of the four statistical problems, we focus on three mainstream ensemble of trees methods: random forests (RF) [19], extreme gradient boosting (XGBoost) [20], and Bayesian additive regression trees (BART) [21] that have generated a wide array of applications in recent years. We will first provide an overview of the fundamentals of the methodologies. Through case studies of two health data examples, we will then illustrate the use of these methods with emphasis on the practical utility of these methods in public health investigations. Finally, we will conclude with a discussion and describe other potential uses of tree-based methods for addressing relevant and emergent health research questions.

## 2. Review of Methods

Tree-based data mining techniques began with classification and regression trees (CART), developed by Breiman and colleagues [22]. It is well known that a single tree is unstable for purposes of prediction, and an ensemble of trees substantially improves the prediction accuracy by reducing the variability of the prediction. The ensemble of three tree methods all use CART as the building block, and each has a different way of “ensembling” trees. We briefly overview each method.

### 2.1. CART

The CART algorithm uses recursive binary splitting to partition the predictor space into non-overlapping homogeneous subsets, referred to as leaves or terminal nodes. In this context, homogeneity is a measure of node purity. A pure node contains observations from a single class. The recursive binary splitting begins at the top node, i.e., the top of the tree, and then successively splits the predictor space. A tree is grown upside down from the top node and terminal nodes. A split of a predictor produces two child nodes. A node of a tree that has child nodes is an internal node. The parts of the tree that connect the nodes are branches. Because the node-splitting process can be depicted in a tree diagram, the method is known as a decision tree method. To predict an individual’s outcome, we can drop the predictors of the individual from the top of a decision tree and assign the individual to the terminal node to which he or she belongs. The predicted outcome for this individual will be the mode (qualitative outcome) or the average (quantitative outcome) of the resulting terminal node.

**Figure 1:**
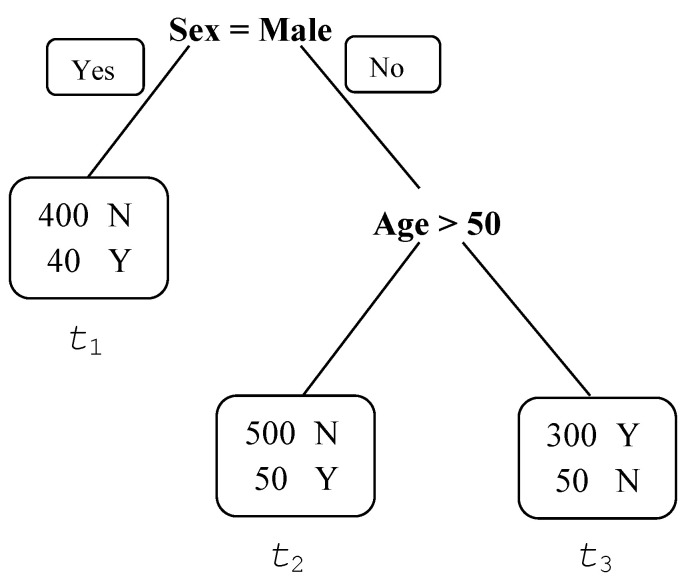
An illustrating classification tree diagram. Y indicates a case and N indicates a non-case.

Figure 1 shows an artificial example to illustrate a decision tree for a binary outcome. The top node is sex; the tree has one internal node, age, and three terminal nodes, $t_{1}=$ Male, $t_{2}=\left\{\mathrm{Female},\mathrm{Age}50\right\}$ and $t_{3}=\left\{\mathrm{Female},\mathrm{Age}\leq 50\right\}$. A female older than 50 will be predicted to be a case because the majority of the individuals in $t_{2}$ are cases, i.e., the mode of $t_{2}$ is a case.

A classification tree is used to predict a qualitative outcome, and a regression tree is used to predict a quantitative outcome. For a classification tree, the CART algorithm uses the Gini index (or cross-entropy) as the criterion for node splitting. The predictor that would result in the biggest decrease in the Gini index will be selected for splitting from all candidate predictors considered for a split at a time. The recursive binary splitting successively splits each of the two child nodes produced by the previous split until some stopping criterion is met. To build an optimal decision tree, the CART approach first grows a large tree and then prunes it down to the optimal size minimizing some cost complexity criterion. The final optimal tree is used for prediction. Growing a regression tree is similar to growing a classification tree. Just as in the classification setting, we use recursive binary splitting to grow a regression tree. However, in the regression setting, an alternative to the Gini index, the residual sum of squares (RSS) is used as a criterion for making the binary splits. That is, the goal is to find splitting rules that minimize the RSS. Section S1 of the Supplemental Materials provides a description of the technical details of the tree-building procedure. It is easy to fit and prune CART trees using the *R* package rpart.

### 2.2. Random Forest

CART trees generate highly interpretable results, but can have high variance in the sense that small changes in data can induce big changes in the resulting tree structure, consequently leading to imprecise prediction. The reason for this is that the goal of the CART algorithm is to segment the covariate space into rectangular regions that contain homogeneous outcomes. If the covariate–response relationship cannot be well characterized by these rectangles, then the CART model will have inaccurate predictions. To tackle these issues, ensemble methods using the CART algorithm as a building block have sprung up over the past two decades.

Bagging, short for bootstrap aggregation, was first developed by Breiman as one of the earliest ensemble techniques [23]. Bagging uses bootstrapping together with the CART algorithm to build an ensemble. Bagging is conceptually simple and the algorithm essentially involves two steps for each of the *B* bootstrap iterations: (1) draw a bootstrap sample from the training data, (2) grow an unpruned classification or regression tree on this sample. Each unpruned tree in the ensemble can be used to predict the outcome of a new sample: class membership for a classification tree and the mean for a regression tree. The prediction for the new sample from the ensemble is the mode of these *B* predicted classes for a qualitative response and is the overall mean of these *B* predicted means for a quantitative response.

Due to the bootstrap resampling technique, bagging supplies out-of-bag (OOB) error for measuring predictive accuracy of the bagged model. At each iteration of bootstrapping, certain samples are left out and not used for fitting the tree model in that iteration. These samples are called OOB samples and can be used to evaluate the predictive performance of the tree model in that iteration. In this way, we can record *B* performance measures from the *B* bootstrapped samples. Averaging the *B* measures over the entire ensemble yields the OOB error.

Bagging generates a distribution of trees, each constructed on a bootstrap sample. The bootstrapped trees may share common structures (e.g., level of nodes, split values, etc.) if the covariate–response relationship can be well modeled by a CART tree. Common structure among a distribution of trees induces tree correlations and consequently prohibits a bagged model from ideally reducing variance of predicted outcomes. To reduce correlation among bootstrapped trees, randomness needs to be added into the tree-building algorithm. Breiman [19] unified an algorithm called RF, which considers a random subset of predictors for each split in the tree-building process on each bootstrap sample. By randomly selecting a smaller set of predictors to be considered for each split, RF prevents the same strong predictors from being repeatedly selected for constructing bootstrap trees. The introduction of randomness into the bagging algorithm reduces correlation among the bootstrap trees and aggregating over decorrelated trees lowers the variance of the predicted outcomes.

RF can be implemented using the *R* package randomForest. Two key tuning parameters for the RF algorithm are the number of randomly selected predictors mtry and the number of trees ntree. From a practical perspective, a larger number of trees elevates the computational burden. It is recommended that ntree=1000 is a good start [24].

### 2.3. Boosting

The idea of boosting is that a weak learner or classifier that predicts outcomes only marginally better than random guessing is boosted into a strong learner with better prediction accuracy. Unlike the RF model in which a separate decision tree is fit to each bootstrap sample of the original training data set and then all of the trees are combined to create a single prediction model, boosting does not use bootstrap sampling but rather the decision trees are grown sequentially, with each tree fit on a modified version of the original data set. For a quantitative outcome, the key steps of boosting are as follows: (1) start a shallow tree $\hat{f}\left(x\right)$ and compute the residuals $r_{i}$, for example, set $\hat{f}\left(x\right)=0$ and $r_{i}=y_{i}$ for all *i* in the training set; (2) fit a tree ${\hat{f}}^{b}$ to the training data with the residuals $r_{i}$ as the response and covariates *X*; (3) update $\hat{f}$ by adding in a shrunken version of the new tree ${\hat{f}}^{b}$ in step (2) $\hat{f}=\hat{f}+\lambda {\hat{f}}^{b}$, where 0λ1; (4) update the residuals $r_{i}$; (5) repeat step (2)–(4) *B* times; (6) sum up all shrunken versions of the trees in previous steps to obtain the boosted model $\hat{f}=\sum_{b=1}^{B}\lambda {\hat{f}}^{b}\left(x\right)$. Specific weights are attached to the terminal nodes of each of the *B* boosted trees, and by summing up the weight scores across the *B* trees for a given *x* we can make a prediction using the boosted model.

The important work by Friedman et al. [25] placed boosting in a statistical framework that ties boosting to a forward stagewise additive model (see step 6) that minimizes a loss function, which measures the distance between the observed and predicted outcomes. Boosting works in a similar way for quantitative and qualitative outcomes but the additive model minimizes different loss functions.

A boosting algorithm that has gained wide popularity in recent years is extreme gradient boosting (XGBoost) [20]. The XGBoost algorithm modifies the loss function of the traditional gradient boosting by including a penalty term for model complexity. In addition, in each step of tree boosting, a random subset of predictors are selected to split to further prevent overfitting. A more detailed description of the XGBoost algorithm is provided in Chen and Guestrin [20].

The XGBoost models can be fitted using the xgboost function in the *R* package xgboost. The key tuning parameters for XGBoost are the shrinkage parameter λ, the number of trees *B*, interaction depth controlling the complexity of the boosted ensemble, and the column subsampling proportion.

### 2.4. BART

BART is a nonparametric Bayesian approach using regression trees. BART is a Bayesian sum-of-trees model with a regularizing prior to keep the individual tree effects small so as to prevent overfitting. Consider a quantitative outcome. To estimate f(x) from models of the form y=f(x)+ϵ, $\epsilon ∼N(0,\sigma^{2})$, BART uses a sum of *m* regression trees $f\left(x\right)=\sum_{j=1}^{m}g\left(x;T_{j},M_{j}\right)$, where $T_{j}$ is the *j*th decision tree structure and $M_{j}$ is the vector of terminal node parameters associated with $T_{j}$, each representing the mean response of the subgroup of observations that fall in that node. The number of trees *m* is usually fixed at a large number, e.g., m=50 or m=200. For a qualitative outcome, probit regression can be used. For example, P(Y=1|X=x)=Φ(f(x)), where Φ is the standard normal cumulative distribution function, and f(x) can be estimated by BART. BART prevents overfitting in the spirit of boosting, but different than boosting which uses a shrinkage parameter to add a small tree each time to previously grown trees, BART uses a regularization prior which holds back the fit of each $\left(T_{j},M_{j}\right)$ tree allowing each to contribute only a small part to the overall fit. The $\left(T_{j},M_{j}\right)$ and σ are treated as parameters in a formal statistical model rather than just algorithmically. A prior is put on the parameters, and the posterior is computed using Markov chain Monte Carlo (MCMC). The algorithm searches for a good f(x), with each tree $g(x;T_{j},M_{j})$ attempting to capture model fit not realized by the others. The prediction can be obtained by drawing values from the posterior distribution of f(x). Because BART is formalized in a statistical model, given the use of the prior, Bayesian posterior measures of uncertainty are readily available.

BART can be implemented using the *R* package BART. It has been shown that the predictive performance of BART using the default prior is highly competitive with other methods that rely on cross-validation to tune algorithm parameters [21,26]. For a qualitative outcome, some pointed out that BART may be sensitive to the mean shrinkage parameter *k*, the optimal value of which can be chosen via cross-validation [27].

## 3. Utilities of Tree-Based Methods

### 3.1. Variable Selection

The foundation of variable selection is the modeling of the covariate–outcome relationship. Traditionally, parametric models have been used to describe how the covariates are related to the outcome via exact functional forms, and variables can be selected in different ways; for example, hypothesis tests between nested models or shrinkage methods that optimize a likelihood penalized for model complexity. Misspecification of these parametric forms can lead to undesirable variable selection results such as noise predictors being selected or important predictors being left out. A large body of work has demonstrated that using flexible tree-based machine learning models can lead to more accurate variable selection results [5,6,17,28,29].

A variable selection method using the OOB variable importance score of an RF model has been used widely in the biomedical research [17,30]. This method selects important predictors based on a recursive elimination procedure. Starting from a full model including all candidate predictors, a sequence of RF models are built. In each iteration, a fraction of predictor variables with the smallest variable importance scores are discarded and a new RF model is built. The OOB error rates from and the minimum error rate of all fitted RF models are recorded. Finally, the set of variables from the RF model that has the minimum number of variables is selected, whose OOB error rate is within one standard error of the overall minimum error rate.

Hu et al. [17] proposed the usage of the recursive feature elimination method with XGBoost. Because XGBoost is not based on bootstrap resampling and does not provide OOB error rate, Hu et al. proposed to use the model classification error for a qualitative outcome and root mean squared error for a quantitative outcome on a 50% hold-out set if the data set is large (e.g., n1000), and use cross-validated errors if the data set is small (e.g., n=350).

Variable selection using BART is based on permutation [28]. The BART model outputs the “variable inclusion proportions” of each predictor variable that represents the relative importance of each predictor. Then by the permutation-based approach, *P* permutations of the response vector are created. The BART model will then be fitted to each of the permuted response vectors and the original predictor variables; and the variable inclusion proportions for each predictor from each BART run are retained and are referred to as the “null” distribution of each predictor’s variable inclusion proportion. A predictor $X_{k}$ is selected if its variable inclusion proportion obtained from the unpermuted data is above the 1−α quantile of the permutation “null” distribution of its variable inclusion proportion. The α is conventionally set at 0.05 or 0.1, and cross-validation can be conducted to determine the optimal value of α.

### 3.2. Counterfactual Prediction

Tree-based machine learning techniques have been adapted into causal inference in recent years and have been shown to produce more accurate treatment effect estimates for their enhanced modeling flexibility that reduces reliance on modeling assumptions [2,31,32,33].

Many causal methods for observational data involve fitting a model for the treatment assignment mechanism (propensity score weighting), a model for the outcome conditional on the treatment and confounding covariates (modeling of the response surface), or both (doubly robust methods). It has been shown that using machine learning can improve propensity score weighting [31,34], which we will demonstrate in the next section, and using highly flexible machine learning methods can improve the estimation of causal effects by precise modeling of the response surface. The conditional models of the response surface can further be used to explore and estimate the treatment effect heterogeneity and identify subgroups that may experience enhanced or reduced treatment effect than the population average [12,35].

We focus on the estimation of average treatment effect, though it is straightforward to compute other causal estimands such as the average treatment effect on the treated. By precisely modeling the response surface, the average treatment effect can be computed through the following steps: (1) fit a tree model on the data using both treatment indicator and predictor variables as the covariates. (2) Use the fitted model to predict the counterfactual outcomes under treatment A and under treatment B for the case of multiple treatments. For the binary treatment setting, counterfactual outcomes are predicted under treatment and under control. (3) Contrast the average of the counterfactual outcomes between the two different treatments and get the estimation of the average treatment effect. Note that in step (2), the counterfactual prediction under treatment A is operated by first setting treatment label for all individuals in the sample population to A and then predicting outcomes for data with treatment A and *X* as the covariates. In step (3), the contrast can be the difference between or the ratio (for a qualitative outcome) of two group means. For BART, the counterfactual prediction is based on the average of posterior draws.

### 3.3. Propensity Score Weighting

Tree-based machine learning can improve the accuracy of propensity score estimation, which in turn improves the estimation of causal effects via inverse probability of treatment weighting. The weighting methods attempt to obtain an unbiased estimator for treatment effect in a way akin to how weighting by the inverse of the selection probability adjusts for unbalances in sampling pools, introduced in survey research [36]. A challenge with weighting is the presence of extreme propensity scores that are close to zero or one, which can result in extreme weights and yield erratic causal estimates with large sample variances. This issue is increasingly likely as the number of treatments increases or as the number of follow up time points increases in a longitudinal study [34]. Machine learning can help reduce the extreme weights and alleviate the adverse impact of extreme weights on causal effect estimation.

### 3.4. Missing Data

Missing data are a pervasive problem in health data sets and pose a substantial challenge for targeted statistical analyses such as variable selection or causal effect estimation. Imputation is widely used for missing at random data. A well-known imputation method is mice [37], by which each incomplete variable is in turn conditioned on all other variables and imputations are drawn from the conditional distributions. The mice uses parametric imputation (conditional) models which may be susceptible to model misspecification biases. The tree-based imputation method missForest [38] employs a similar chained equation approach for imputation as implemented in mice but uses RF models for the conditional distributions regressing each incomplete variable against all other variables. It has been shown that missForest has a better imputation performance than mice when the true data dependence structures among the variables are nonlinear [39]. BART has been utilized for the sequential imputation of missing covariates [40], and XGBoost was recently used for multiple imputation; however, there is either a lack of easy-to-implement software or a published reference for these two works.

## 4. Case Studies of Tree-Based Methods

### 4.1. Confounder Selection

We demonstrate how variable selection can be performed by three tree-based machine learning methods. We apply each of the methods to the clinical encounter and medicare claims data on 11,980 patients with stage I–IIIA non-small cell lung cancer (NSCLC) drawn from the Surveillance, Epidemiology, and End Results (SEER)-Medicare database. These patients were above 65 years of age, diagnosed between 2008 and 2013 and underwent surgical resection via one of three approaches: robotic-assisted surgery, video-assisted thoracic surgery, or open thoracotomy. The data set contains individual-level information at baseline on the following variables: age, gender, marital status, race, ethnicity, income level, comorbidities, cancer stage, tumor size, tumor site, cancer histology, and whether they underwent positron emission tomography, chest computed tomography, or mediastinoscopy. A detailed description of patient characteristics can be found in Hu et al. [2] and is also provided in Table S1 of the Supplemental Materials. There were a total of 14 potential predictor variables.

Drawing causal inference from nonexperimental data involves adjusting for confounders. A variable is a confounder if it predicts both treatment and outcome. Confounder selection is critical for the estimation of causal effect [41]. We illustrate the selection of variables important to the outcome. Variables relevant to the treatment assignment mechanism can be selected in a similar fashion. We use the presence of respiratory complication within 30 days of surgery or during the hospitalization in which the primary surgical procedure was performed as the outcome. The respiratory complication rate was 30.1% in the robotic-assisted surgery group, 33.6% in the video-assisted thoracic surgery group, and 33.3% in the open thoracotomy group.

Table 1 displays variable selection results from each of three tree-based machine learning methods. The BART method selected the most number (five) of predictors, and XGBoost and RF selected relatively fewer predictors, two and three, respectively. To see how well the selected variables predict the outcome, we also present in Table 1 the five-fold cross-validated area under the receiver operating characteristics curve (AUC) for each of three models with the selected predictors. BART delivered a higher AUC of 0.85 than XGBoost and RF, which produced similar AUCs between 0.7 and 0.75.

**Figure 2:**
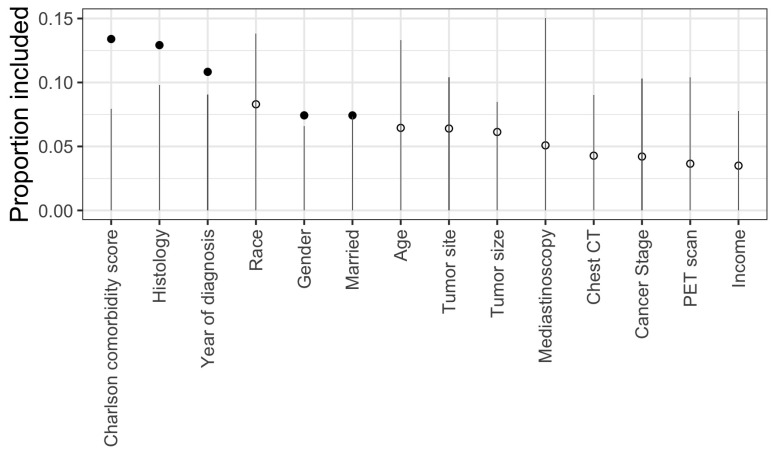
Visualization of the BART variable selection algorithm. The vertical lines are the threshold levels determined from the “null” distributions for variable inclusion proportions computed from 100 permutated data. Variable inclusion proportions from the original (unpermutated) data passing this threshold are displayed as solid dots. Open dots correspond to variables that are not selected.

Figure 2 visualizes the variable selection process via BART. For each potential predictor variable, the selection threshold determined by the “null” distribution of its variable inclusion proportion is represented by the vertical line. If a variable’s variable inclusion proportion on the original unpermutated data exceeds the threshold, then the variable is selected and represented by a solid dot; otherwise an open dot. Indicated also in Figure 2 is the rank of importance of each predictor variable. Among the five selected predictors, the Charlson comorbidity score appeared to be the most important predictor and the demographic information such as gender and marital status were less important. By contrast, RF and XGBoost use a recursive backward elimination procedure for variable selection based on the variable importance score. XGBoost ranked age as the most important predictor and RF selected histology. The complete list of variable importance scores are presented in Table S3.

### 4.2. Comparative Effectiveness Analysis

Shown in Table 2, we now apply the tree-based methods to the SEER-Medicare data to estimate the comparative treatment effects of the three surgical approaches on postoperative respiratory complications. We show the treatment effects on the basis of relative risk. For the purposes of illustration, we included all 14 confounders in modeling the response surface. The causal effect estimates suggest that judging by postoperative respiratory complications, there was not a surgical approach that led to significantly better outcomes. Note that because BART is based on a Bayesian probability model, the uncertainty intervals about the treatment effect estimates can be easily obtained from the posteriors [10]. For RF and XGBoost, we provided the confidence intervals using nonparametric bootstrapping, but the theoretical justification warrants further research.

### 4.3. Propensity Score Weight Estimator

Propensity score weighting is another causal inference method to estimate the causal effects. The key step is the accurate estimation of the inverse probability of treatment weights to reduce selection bias. We apply tree-based methods to the SEER-Medicare data to estimate the weights. In the treatment assignment model, the response variable was treatment, which in our case has three groups, and the covariates were the 14 confounders. To estimate the weights using RF and XGBoost, we fit a multinomial logit model,

$$\begin{array}{cc} \log\left\{\frac{P(T=1|x)}{P(T=3|x)}\right\} & =f_{1}\left(x\right)\\ \log\left\{\frac{P(T=2|x)}{P(T=3|x)}\right\} & =f_{2}\left(x\right), \end{array}$$

where *T* indicates treatment groups, *x* represents the 14 confounders, and $f_{1}$ and $f_{2}$ are to be estimated by RF and XGBoost. BART uses multinomial probit regression for categorical variables. To estimate the weights using BART, we fit a multinomial probit regression model represented in terms of a latent variable model:

$$\begin{array}{c} T_{1}^{*}=f_{1}\left(x\right)+\varepsilon_{1}\\ T_{2}^{*}=f_{2}\left(x\right)+\varepsilon_{2}\\ T_{3}^{*}=f_{3}\left(x\right)+\varepsilon_{3}, \end{array}$$

where ε∼N(0,Σ), and then

$$T=\left\{\begin{array}{cc} 1 & \;\mathrm{if}\;T_{1}^{*}T_{2}^{*},T_{3}^{*}\\ 2 & \;\mathrm{if}\;T_{2}^{*}T_{1}^{*},T_{3}^{*}\\ 3 & \;\mathrm{if}\;T_{3}^{*}T_{1}^{*},T_{2}^{*} \end{array}\right.,$$

where $f_{1}$, $f_{2}$, and $f_{3}$ are to be estimated by BART. The weights were then calculated as the inverse of treatment probabilities. For RF and XGBoost,

$$\mathrm{weights}=\left\{\begin{array}{cc} 1+\exp\left[f_{1}\left(x\right)\right]+\exp\left[f_{2}\left(x\right)\right]/\exp\left[f_{1}\left(x\right)\right] & \;\mathrm{for}\;\mathrm{treatment}\;T=1\\ 1+\exp\left[f_{1}\left(x\right)\right]+\exp\left[f_{2}\left(x\right)\right]/\exp\left[f_{2}\left(x\right)\right] & \;\mathrm{for}\;\mathrm{treatment}\;T=2\\ 1+\exp\left[f_{1}\left(x\right)\right]+\exp\left[f_{2}\left(x\right)\right] & \;\mathrm{for}\;\mathrm{treatment}\;T=3\;. \end{array}\right.$$

**Figure 3:**
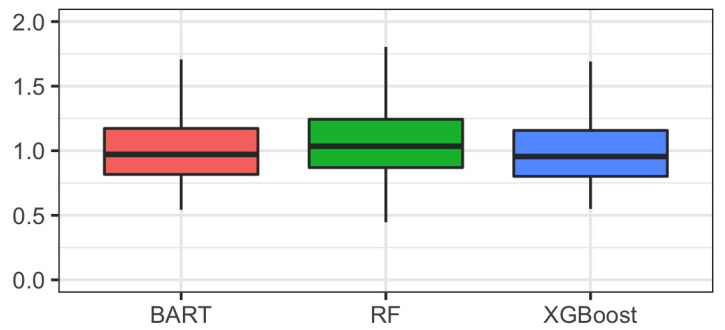
Distributions of the inverse probability of treatment weights estimated by BART, random forest, and XGBoost.

Figure 3 shows the distribution of the inverse probability of treatment weights estimated by each of three tree-based methods. Note that the posterior mean of the weights was used for BART. The weights estimated from the three methods had similar distributions and there were no extreme weights that were of concern.

### 4.4. Handing Missing Data

In this section, we demonstrate how the RF-based method missForest can be used to impute missing values of covariates and/or outcomes under the missing at random mechanism, using the data set from the Study of Women’s Health Across the Nation (SWAN) [17]. The SWAN study was a multicenter, longitudinal study aiming to understand women’s health across the menopause transition. The analysis data set included 3302 women aged between 42 and 52, who were enrolled in 1996–1997 from seven sites of the US and were followed to 2018 annually. There is a strong interest in using the SWAN data to identify key risk factors for health outcomes such as metabolic syndrome [8,17]. However, a challenging issue is the presence of missing data. Among 60 potential predictor variables, only 11 variables were fully observed; the amount of missing data in the variables ranged from 0.1% to 27.1%. A detailed description of this data set can be found in Hu et al. [17].

Under the missing at random mechanism, a popular statistical technique for handling missing data is imputation. For example, imputation was combined with tree-based variable selection methods to identify key predictors for metabolic syndrome [17]. We used missForest to impute the missing values in predictor variables. To begin, an initial guess (e.g., the mean value) was made for the missing values in X. Then, predictor variables were sorted according to the amount of missing values starting with the lowest amount. For each incomplete variable, the missing values were imputed by first fitting an RF with the incomplete variable as the outcome and all other variables as covariates and then predicting the missing values from the trained RF. The imputation procedure was repeated until the difference between the newly imputed data matrix and the previous one increased for the first time with respect to both continuous variables and categorical variables [38].

**Figure 4:**
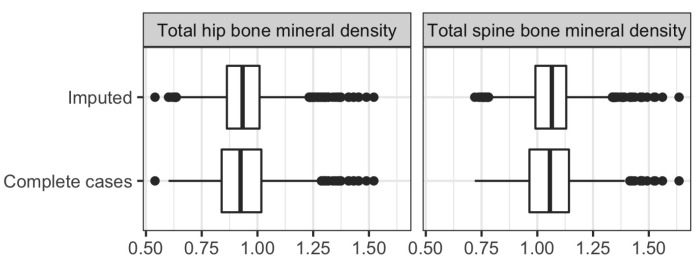
A comparison of the distributions of values for total hip bone mineral density and total spine bone mineral density among the imputed values and among the complete cases.

Figure 4 shows, for two continuous variables that had the largest missingness proportions: 26.6% in total hip bone mineral density and 27.1% in total spine bone mineral density, the distribution of values among the complete cases (with missing records discarded) and among the imputed values. There is no appreciable difference between the two distributions for either variable. A closer look at the summary statistics of the distributions in Table S4 conveys a similar message.

## 5. Discussion

We provided an overview of three key tree-based machine learning methods that have gained popularity in biomedical studies and demonstrated how these methods can be leveraged to solve important research questions in four domains: variable selection, estimation of causal effects, propensity score weighting and missing data. We illustrated that the central idea of using ensemble tree methods for these research questions is accurate prediction via flexible modeling.

Through four data examples—one for each research domain—we demonstrated the utility of tree-based machine learning methods in health studies. We elucidated how modern tree-based variable selection methods can be used to identify important predictors for patient-oriented health outcomes such as postoperative respiratory complications for lung cancer patients. Variable selection can also be useful for identifying critical confounding variables for causal analyses using nonexperimental data. Tree-based methods were applied to a large-scale SEER-Medicare data set to estimate the comparative treatment effectiveness of multiple treatment options. The elevated modeling flexibility offered by tree-based methods leads to more accurate effect estimates via more accurate modeling of the response surface. Another popular causal inference technique, propensity score weighting, can also benefit from the flexibility of tree-based models. Finally, we used the SWAN study data to exposit how random forest can be used to better impute the missing data that were present in multiple variables of different data types. The imputed data set can be turned over for whatever kind of analysis would be applied to a complete data set.

The performance of the tree-based methods when applied to each of the four domains can be evaluated via simulation using domain-specific criteria. For variable selection, methods can be judged based on the ability of selecting most useful predictors and least noise predictors. For example, precision, recall, $F_{1}$ score, and type I error have traditionally been used as the performance metrics for variable selection methods [17,28]. When used to estimate the causal treatment effects, the bias and root mean squared error in the effect estimates can be used to assess the methods performance [2]. How machine learning can improve the estimation of the inverse probability weights can be evaluated by checking the distribution of the estimated weights, e.g., whether there are spiky weights [13]. Finally, for missing data imputation, the bias and root mean squared error in the estimated values can be used to compare the performance of the methods [40].

There are other important research questions we can address by leveraging the ensemble tree methods. For example, relevant to precision medicine, tree-based methods can facilitate ascertaining subpopulations who may experience enhanced or reduced treatment effect than population average [12,35]. Moreover, as a recommended strategy to examine how sensitive the estimated treatment effect is to potential unmeasured confounding, the tree-based machine learning technique BART has been used to develop a well-performing sensitivity analysis method [18]. It is worthwhile to further expand the use of ensemble tree methods in health research.

## 6. Conclusions

Through examples of identifying important risk predictors, estimating the causal treatment effects, estimating the inverse probability weights for propensity score weighting analysis and imputing the missing data, tree-based methods are a flexible tool and their proven prediction accuracy can help improve analysis approaches in health investigations.

## Figures and Tables

**Table 1:** Variables selected by each method, and 5-fold cross-validated area under the receiver operating characteristics curve using each model with selected variables.

Methods	Selected Variables	AUC
BART	Chalson comorbidity score, gender, married, histology, year of diagnosis	0.85
XGBoost	Age, year of diagnosis	0.72
RF	Chalson comorbidity score, histology	0.74

**Table 2:** Causal inferences about average treatment effects of three surgical approaches on postoperative respiratory complications based on the relative risk, using the SEER-Medicare lung cancer data. The 95% uncertainty intervals are displayed in parentheses. All 14 potential confounders were used. RAS: robotic-assisted surgery; VATS: video-assisted thoracic surgery; OT: open thoracotomy.

Methods	RAS vs. OT	RAS vs. VATS	OT vs. VATS
BART	0.94 (0.72, 1.16)	1.09 (0.84, 1.34)	1.12 (0.87, 1.37)
XGBoost	0.91 (0.64, 1.13)	1.04 (0.79, 1.28)	1.08 (0.84, 1.33)
RF	0.90 (0.63, 1.14)	1.03 (0.78, 1.29)	1.06 (0.82, 1.35)

## Data Availability

*R* codes for implementing all methods are provided in Section S3 of the Supplementary Materials. This study used the deidentified SEER-Medicare database and SWAN data. The SEER-Medicare data are available upon approval of data requests by the Information Management Services of the National Cancer Institute. The SWAN data are publicly available at https://www.swanstudy.org/swan-research/data-access, accessed on 15 October 2022.

## Supplementary Materials

The following supporting information can be downloaded at: https://www.mdpi.com/article/10.3390/ijerph192316080/s1.
