# Supplementary material for: Using Tree-Based Machine Learning for Health Studies: Literature Review and Case Series
Source: Int J Environ Res Public Health. 2022 Dec 1;19(23):16080. doi: 10.3390/ijerph192316080 (PMC9736500; doi:10.3390/ijerph192316080)
Supplement: Supplementary file 1 [file ijerph-19-16080-s001.zip › ijerph-2000981-supplementary.pdf]

Supplement to "Using Tree-based Machine Learning for Health Studies: Literature Review and Case Series"

Liangyuan Hu[1] and Lihua Li[2]

[1]Department of Biostatistics and Epidemiology, Rutgers University School of Public Health, USA

[2]Department of Population Health Science and Policy, Icahn School of Medicine at Mount Sinai, USA

1 Recursive binary splitting, pruning and prediction for CART

We use the Gini index to illustrate the recursive binary splitting of the decision tree. The Gini index measures how pure a node is. If all observations in a node are from the same class, then the node is pure. For a two-class problem, the Gini index is defined as $G = \hat{p}_1(1-\hat{p}_1)+\hat{p}_2(1-\hat{p}_2) = 2\hat{p}_1\hat{p}_2$, where $\hat{p}_1$ is the proportion of class 1 in a node, and $\hat{p}_2$ is the proportion of class 2 in a node. If the class proportions are close to one or zero – indicating a pure node – then the Gini index has a very small value. The Gini index is used in the CART algorithm to guide node splitting. Decrease in the Gini index from a split is desired; priority will be given to the predictor that would generate the biggest decrease from a split. A split over sex results in a contingency table shown as Web Table 1.

The split over sex produces two child nodes, male and female. The Gini index before the split is $2(390/1340)(950/1340) = .413$. After the split, the index can be calculated within

Web Table 1: Contingency table illustrating Gini index associated with split on Sex

	Class 1 (Y)	Class 2 (N)	Total
Male	40	400	440
Female	350	550	900
Total	390	950	1340

each of the two child nodes. The value is $2(40/440)(400/440) = .165$ within the male node and $2(350/900)(550/900) = .475$ within the female node. The overall Gini index for this split is the weighted average of the two values, each weighted by the sample proportion of the child nodes, $.165(440/1340) + .475(900/1340) = .374$. The split over sex yields a decrease of $.039(.413 - .039)$ in the Gini index. The node splitting procedure continues with each of the two child nodes until some stopping criterion is met. Examples of stopping rules include a pre-defined minimum number of samples in a node and the maximum number of terminal nodes. When a continuous or categorical variable is considered for a split, the samples are first sorted based on the predictor values, then a partitioning algorithm is used to search for the optimal split value that minimizes the Gini index associated with the split. Trees grown to have maximum terminal nodes may over-fit the data, but a small tree is unlikely to capture the full scope of the data. A commonly used strategy for finding the optimal tree size is by means of cost complexity tuning. This process is referred to as tree pruning. The tree pruning process can be described as follows: first, grow a large tree reaching a predefined number of terminal nodes. Then find a subtree T with $|T|$ number of terminal nodes, in which the prediction accuracy is penalized by the tree size $|T|$. The subtree T with optimal tree size should minimize the cost complexity criterion: $C_\alpha(T) = E + \alpha|T|$, where E is the total classification error in each

terminal node and α is the complexity parameter controlling the tradeoff between the tree size $|T|$ and prediction accuracy of the tree E. To find the optimal tree, we first need to obtain optimal value for the complexity parameter $\hat{\alpha}_{\text{opt}}$. First, for a given value of α, we obtain an optimal subtree minimizing the cost complexity criterion $C_\alpha(T)$. Second, we obtain the K-fold cross-validation classification error for this tree. We repeat the two steps for a sequence of α values to generate a sequence of classification errors, and then the optimal $\hat{\alpha}_{\text{opt}}$ is the value of α that corresponds to the smallest classification error. The optimal subtree for $\hat{\alpha}_{\text{opt}}$ is the final optimal pruned tree and can be used for prediction.

Growing a regression tree is similar to growing a classification tree. Just as in the classification setting, we use recursive binary splitting to grow a regression tree. However, in the regression setting, alternative to Gini index, residuals sum of squares (RSS) are used as a criterion for making the binary splits. That is, the goal is to find splitting rules that minimize the RSS,

$$\sum_{j=1}^{J} \sum_{i \in R_j} (y_i - \hat{y}_{R_j})^2,$$

where $R_1, \ldots, R_J$ are the J distinct and non-overlapping subpaces of the covariate space X, or the end notes. Using the illustrating example shown in Figure 1, $R_1 = \{X|\text{Sex} = \text{Male}\}$, $R_2 = \{X|\text{Sex} = \text{Female and age} \leq 50\}$, and $R_3 = \{X|\text{Sex} = \text{Female and age} > 50\}$.

2 Additional tables and figures

Web Table 2: Baseline patient characteristics in SEER-Medicare data.

Characteristics	Robotic-Assisted Surgery $N = 396$	Video-assisted thoracic surgery $N = 6582$	Open Thoracotomy $N = 5002$
Age (years), mean (SD)	74.3 (5.7)	73.9 (5.4)	74.5 (5.7)
Female, N (%)	223 (56.3)	3446 (52.4)	2941 (58.8)
Married, N (%)	227 (57.3)	3753 (57.0)	2802 (56.0)
Race, N (%)			
White	320 (80.8)	5694 (86.5)	4369 (87.3)
Black	21 (5.3)	364 (5.5)	248 (5.0)
Hispanic	15 (3.8)	218 (3.3)	139 (2.8)
Other	40 (10.1)	306 (4.6)	246 (4.9)
Median household annual income, N (%)			
1st quartile	97 (24.5)	2132 (32.4)	1009 (20.2)
2nd quartile	88 (22.2)	1729 (26.3)	1193 (23.9)
3rd quartile	98 (24.7)	1345 (20.4)	1143 (22.9)
4th quartile	113 (28.5)	1376 (20.9)	1657 (33.1)
Charlson comorbidity score, N (%)			
$0 - 1$	154 (38.9)	2163 (32.9)	1810 (36.2)
$1 - 2$	113 (28.5)	1944 (29.5)	1379 (27.6)
> 2	129 (32.6)	2475 (37.6)	1813 (36.2)
Year of diagnosis, N (%)			
2008-2009	14(3.5)	2686 (40.8)	1484 (29.7)
2010	33 (8.3)	1123 (17.1)	857 (17.1)
2011	85 (21.5)	1033 (15.7)	866 (17.3)
2012	131 (33.1)	899 (13.7)	821 (16.4)
2013	133 (33.6)	841 (12.8)	974 (19.5)
Cancer stage, N (%)			
Stage I	295 (74.5)	4195 (63.7)	3884 (77.6)
Stage II	63 (15.9)	1504 (22.9)	709 (14.2)
Stage IIIA	38 (9.6)	883 (13.4)	409 (8.2)
Tumor size, in mm, N (%)			
≤ 20	160 (40.4)	1967 (29.9)	2232 (44.6)
$21 - 30$	98 (24.7)	1696 (25.8)	1388 (27.7)
$31 - 50$	109 (27.5)	1804 (27.4)	987 (19.7)
≥ 51	29 (7.3)	1084 (16.5)	367 (7.3)
Histology, N (%)			
Adenocarcinoma	255 (64.4)	3757 (57.1)	3348 (66.9)
Squamous cell carcinoma	107 (27.0)	2165 (32.9)	1167 (23.3)
Other histology	34 (8.6)	660 (10.0)	487 (9.7)
Tumor site, N (%)			
Upper lobe	215 (54.3)	3829 (58.2)	2859 (57.2)
Middle lobe	27 (6.8)	308 (4.7)	335 (6.7)
Lower lobe	141 (35.6)	2195 (33.3)	1720 (34.4)
Other site	13 (3.3)	250 (3.8)	88 (1.8)
PET scan, N (%)	302 (76.3)	5004 (76.0)	3410 (68.2)
Chest CT, N (%)	263 (66.4)	4525 (68.7)	3148 (62.9)
Mediastinoscopy, N (%)	62 (15.7)	715 (10.9)	420 (8.4)

Abbreviations: PET = positron emission tomography; SD = standard deviation; CT = computer tomography

Web Table 3: Variable importance score for each predictor using XGBoost and Random Forest.

Variable	XGBoost	RF
Age	0.20	0.04
Year of diagnosis	0.12	0.02
Chalson comorbidity score	0.10	0.10
Income	0.09	0.01
Tumor size (nm)	0.08	0.02
Tumor site	0.07	0.04
Histology	0.06	0.12
Cancer stage	0.05	0.06
Race	0.04	0.03
Marriage	0.04	0.02
Gender	0.04	0.01
Chest CT	0.04	0.05
PET Scan	0.03	0.04
Mediastinoscopy	0.02	0.04

Web Table 4: The distribution of values for total hip bone mineral density and total spine bone mineral density among the imputed variables and among the complete cases.

	Min	1st Q	Median	Mean	3rd Q	Max
Total hip bone mineral density						
Complete cases	0.54	0.84	0.92	0.93	1.02	1.52
Imputed	0.54	0.86	0.93	0.94	1.00	1.52
Total spine bone mineral density						
Complete cases	0.72	0.96	1.06	1.06	1.14	1.64
Imputed	0.72	0.99	1.07	1.06	1.13	1.64

3 R code to implement the tree-based methods

3.1 Confounder selection using BART

```
library(tidyverse)
# Read in the data
data <- read_csv("data.csv")
library(bartMachine)
confounder <- data %>%
  select(-Treatment, -respiratory_complications30d) %>%
  as.data.frame()
# Run BART
bartMachine_result <-bartMachine(y = data$respiratory_complications3
    0d %>% as.factor(), X=confounder)
# Run BART variable selection
var_select1 <- var_selection_by_permute(bartMachine_result, plot=
    FALSE)
# Summarize the permutated variable inclusion proportions for each
    variable
vs_permutate_factor <- var_select1$permute_mat %>%
  as_tibble() %>%
  transmute(Age = Age,
            Gender = Gender_Female + Gender_Male,
            Married = Married_No + Married_Yes,
            Race = Race_White + Race_Black + Race_Hispanic + Race_
                Other,
            Income = `Income_1st quartile` + `Income_2nd quartile` +
```

```
     `Income_3rd quartile` + `Income_4th quartile`,
  `Chalson comorbidity score` = `Chalson comorbidity score
    _0 - 1` + `Chalson comorbidity score_1 - 2` + `
    Chalson comorbidity score_> 2`,
  `Year of diagnosis` = `Year of diagnosis_2008-2009` + `
    Year of diagnosis_2010` + `Year of diagnosis_2011` +
    `Year of diagnosis_2012` + `Year of diagnosis_2013`,
  `Cancer Stage` = `Cancer Stage_Stage I` + `Cancer Stage_
    Stage II` + `Cancer Stage_Stage III`,
  `Tumor size` = `Tumor size (nm)_<= 20` + `Tumor size (nm
    )_21 - 30` + `Tumor size (nm)_31 - 50` + `Tumor size
    (nm)_>= 51`,
  Histology = Histology_Adenocarcinoma + `Histology_Other
    hisology` + `Histology_Squamous cell carcinoma`,
  `Tumor site` = `Tumor site_Lower lobe` + `Tumor site_
    Middle lobe` + `Tumor site_Other site` + `Tumor site_
    Upper lobe`,
  `PET scan` = `PET scan_No` + `PET scan_Yes`,
  `Chest CT` = `Chest CT_No` + `Chest CT_Yes`,
  Mediastinoscopy = `Mediastinoscopy_No` + Mediastinoscopy
    _Yes)
# Summarize the original variable inclusion proportions for each
   variable
vs_prop_factor <- var_select1$var_true_props_avg %>%
  as.data.frame() %>%
  t %>%
  as.tibble() %>%
```

```
transmute(Age = Age,
          Gender = Gender_Female + Gender_Male,
          Married = Married_No + Married_Yes,
          Race = Race_White + Race_Black + Race_Hispanic + Race_
             Other,
          Income = `Income_1st quartile` + `Income_2nd quartile` +
              `Income_3rd quartile` + `Income_4th quartile`,
          `Chalson comorbidity score` = `Chalson comorbidity score
             _0 - 1` + `Chalson comorbidity score_1 - 2` + `
             Chalson comorbidity score_> 2`,
          `Year of diagnosis` = `Year of diagnosis_2008-2009` + `
             Year of diagnosis_2010` + `Year of diagnosis_2011` +
             `Year of diagnosis_2012` + `Year of diagnosis_2013`,
          `Cancer Stage` = `Cancer Stage_Stage I` + `Cancer Stage_
             Stage II` + `Cancer Stage_Stage III`,
          `Tumor size` = `Tumor size (nm)_<= 20` + `Tumor size (nm
             )_21 - 30` + `Tumor size (nm)_31 - 50` + `Tumor size
             (nm)_>= 51`,
          Histology = Histology_Adenocarcinoma + `Histology_Other
             hisology` + `Histology_Squamous cell carcinoma`,
          `Tumor site` = `Tumor site_Lower lobe` + `Tumor site_
             Middle lobe` + `Tumor site_Other site` + `Tumor site_
             Upper lobe`,
          `PET scan` = `PET scan_No` + `PET scan_Yes`,
          `Chest CT` = `Chest CT_No` + `Chest CT_Yes`,
          Mediastinoscopy = `Mediastinoscopy_No` + Mediastinoscopy
             _Yes)
```

```
# Calculate the cutoff values using local threshold
localcutoff<-apply(vs_permutate_factor, 2, quantile, probs=0.9)
# Merge the cutoff by var names
cut_mat<-data.frame("localcutoff"=localcutoff, "varnames"=names(
   localcutoff))
prop_mat<-data.frame("prop"=t(vs_prop_factor), "varnames"=names(vs_
   prop_factor))
vs_mat<-merge(cut_mat, prop_mat, by="varnames", all=TRUE)
vs_mat$local_results<-ifelse(vs_mat$prop>vs_mat$localcutoff,1,0)
vs_mat$varnames[vs_mat$local_results==1] # 5 selected
```

3.2 Confounder selection using RF

```
library(varSelRF)
out_vec <- data$respiratory_complications30d %>% as.factor()
rf_varselect <- varSelRF(confounder, out_vec, vars.drop.frac = 0.1)
rf_varselect$selected.vars # Variables selected by RF
```

3.3 Confounder selection using BART

```
library(xgboost)
confounder <- data %>%
  select(-Treatment, -respiratory_complications30d) %>%
  as.data.frame()
metrics_mat<-data.frame(matrix(NA,nrow=ncol(confounder), ncol=1))
names(metrics_mat)<-"met"
model_list<-list(NA)
```

```
var_out_list<-list(NA)
out_vec <- data$respiratory_complications30d
cov_mat0 <- confounder
cov_mat_v0<-data.matrix(confounder)
xgb_v0 <- xgboost(data = cov_mat_v0, label = out_vec, verbose = F,
   missing = NA,objective = "binary:logistic", na.action = "na.pass"
   )
imp_list_i<-list(imp_list)
i<-1
nvar<-ncol(confounder)
# Random split data into training and test for xgb (1:1)
sam_id<-sample(c(1:nrow(confounder)), round(nrow(confounder)/2))
dat_train<-confounder[sam_id,]
dat_test<-confounder[-sam_id,]
out_vec_train<-out_vec[sam_id]
out_vec_test <- out_vec[-sam_id]
class_error<-function(y, yhat, phat=NULL){
  Metrics::ce(actual=y, predicted=yhat)
}
while(nvar>=1){
  cov_mat<-confounder
  cov_mat_train<-dat_train
  cov_mat_test<-dat_test
  if(is.factor(cov_mat)==1){
    # Fit using train
    ff<-~.
    mf_train<-model.frame(formula = ff, data = data.frame(cov_mat_
```

```
    train), na.action = "na.pass")
  names(mf_train)<-names(datax1)[names(datax1)!="event"]
  cov_mat_v1_train<-model.matrix(object = ff, data = mf_train)
  mf_test<-model.frame(formula = ff, data = data.frame(cov_mat_
    test), na.action = "na.pass")
  names(mf_test)<-names(datax1)[names(datax1)!="event"]
  cov_mat_v1_test<-model.matrix(object = ff, data = mf_test)
  xgb_v1 <- xgboost(data = cov_mat_v1_train, label = out_vec_train
    , verbose = F, missing = NA, objective = "binary:logistic",
    na.action = "na.pass", nrounds=nroundx, nthreadx=nthreadx)
  # Predict on test
  phat<-predict(xgb_v1, newdata = cov_mat_v1_test, missing = NA)
  yhat<-factor(as.character(ifelse(phat>=0.5,"Yes","No")), levels
    = c("No", "Yes"))
  metrics_mat$met[i]<-class_error(y=out_vec_test, yhat=as.numeric(
    yhat), phat=phat)
  var_out<-"0"
  var_out_list[[i]]<-var_out
  datax1<-datax1[,-which(names(datax1)%in%var_out)]
}else{
  # Fit using train
  if(is.null(ncol(cov_mat_train))==1){
    cov_mat_train<-data.frame(cov_mat_train)
    cov_mat_test<-data.frame(cov_mat_test)
    names(cov_mat_train)<-names(cov_mat_test)<-names(confounder)
  }
  ff<-~.
```

```
  mf_train<-model.frame(formula = ff, data = data.frame(cov_mat_
     train), na.action = "na.pass")
  cov_mat_v1_train<-model.matrix(object = ff, data = mf_train)

  mf_test<-model.frame(formula = ff, data =  data.frame(cov_mat_
     test), na.action = "na.pass")
  cov_mat_v1_test<-model.matrix(object = ff, data = mf_test)

  xgb_v1 <- xgboost(data = cov_mat_v1_train, label = out_vec_train
     , verbose = F, missing = NA, objective = "binary:logistic",
     na.action = "na.pass", nrounds=200)
  # Predict on test
  phat<-predict(xgb_v1, newdata = cov_mat_v1_test, missing = NA)
  yhat<-factor(as.character(ifelse(phat>=0.5,"Yes","No")), levels
     = c("No", "Yes"))
  metrics_mat$met[i]<-class_error(y=out_vec_test, yhat=as.numeric(
     yhat), phat=phat)
} # end else
if(is.null(ncol(cov_mat))==0){
  num_var_exclude<-round(0.1*ncol(cov_mat))
  if(num_var_exclude==0){num_var_exclude=1}
  imp_list<-imp_list[order(imp_list$Gain, decreasing=FALSE),]
  var_out<-imp_list$Feature[c(1:num_var_exclude)]
  var_out_list[[i]]<-var_out
}else{
  num_var_exclude<-1
  imp_list<-imp_list[order(imp_list$Gain, decreasing=FALSE),]
```

```
    var_out<-imp_list$Feature[c(1:num_var_exclude)]
    var_out_list[[i]]<-var_out
  }
  confounder<-confounder[,-which(names(confounder) %in% var_out),
     drop=FALSE]
  dat_train<-dat_train[,-which(names(dat_train) %in% var_out),drop=
     FALSE]
  dat_test<-dat_test[,-which(names(dat_test) %in% var_out),drop=
     FALSE]
  imp_list_i[[i+1]]<-imp_list<-imp_list[imp_list$Feature %in% var_
     out==0,]
  i<-i+1
  nvar<-nvar-num_var_exclude
}
metrics_mat
```

3.4 Counterfactual prediction using BART

```
library(CIMTx)
data <- data %>%
  mutate(Treatment= case_when(Treatment == "OT" ~ 1,
                              Treatment == "RAS" ~ 2,
                              Treatment == "VATS" ~ 3))
# Fit BART
bart_mod_pred_1 = BART::pbart(x.train = data %>% as.data.frame(), y.
   train = data$respiratory_complications30d, x.test = data %>% as.
   data.frame() %>% mutate(Treatment = 1))
```

```
bart_mod_pred_2 = BART::pbart(x.train = data %>% as.data.frame(), y.
   train = data$respiratory_complications30d, x.test = data %>% as.
   data.frame() %>% mutate(Treatment = 2))
bart_mod_pred_3 = BART::pbart(x.train = data %>% as.data.frame(), y.
   train = data$respiratory_complications30d, x.test = data %>% as.
   data.frame() %>% mutate(Treatment = 3))
mean(bart_mod_pred_2$prob.test.mean)/mean(bart_mod_pred_1$prob.test.
   mean)
mean(bart_mod_pred_2$prob.test.mean)/mean(bart_mod_pred_3$prob.test.
   mean)
mean(bart_mod_pred_1$prob.test.mean)/mean(bart_mod_pred_3$prob.test.
   mean)
```

3.5 Counterfactual prediction using RF

```
library(randomForest)
rf_w_1 <-
  randomForest(
    y = as.factor(
      data$respiratory_complications30d
    ),
    x = data %>%
      select(-respiratory_complications30d) %>%
      as.data.frame(),
    xtest = data %>%
      select(-respiratory_complications30d) %>%
      mutate(Treatment = 1) %>% as.data.frame(),
```

```
    ntree = 2000
  )
rf_w_2 <-
  randomForest(
    y = as.factor(
      data$respiratory_complications30d
    ),
    x = data %>%
      select(-respiratory_complications30d) %>%
      as.data.frame(),
    xtest = data %>%
      select(-respiratory_complications30d) %>%
      mutate(Treatment = 2) %>% as.data.frame(),
    ntree = 2000
  )
rf_w_3 <-
  randomForest(
    y = as.factor(
      data$respiratory_complications30d
    ),
    x = data %>%
      select(-respiratory_complications30d) %>%
      as.data.frame(),
    xtest = data %>%
      select(-respiratory_complications30d) %>%
      mutate(Treatment = 3) %>% as.data.frame(),
    ntree = 2000
```

```
  )
```

3.6 Counterfactual prediction using XGBoost

```
out_vec <- data$respiratory_complications30d
cov_mat0 <- data %>%
    select(-respiratory_complications30d) %>%
    as.data.frame()
cov_mat_v0 <- data.matrix(cov_mat0)
xgb_v0 <- xgboost(data = cov_mat_v0, label = out_vec, verbose = F,
   missing = NA,objective = "binary:logistic", na.action = "na.pass"
   )
cov_mat_w_1 <-
    data.matrix(
      # object = ~ .,
      data %>%
        select(-respiratory_complications30d) %>%
        mutate(Treatment = 1)
    )
phat_w_1 <- predict(xgb_v0, newdata = cov_mat_w_1)
cov_mat_w_2 <-
    data.matrix(
      # object = ~ .,
      data %>%
        select(-respiratory_complications30d) %>%
        mutate(Treatment = 2)
    )
```

```
phat_w_2 <- predict(xgb_v0, newdata = cov_mat_w_2)
cov_mat_w_3 <-
    data.matrix(
      # object = ~ .,
      data %>%
        select(-respiratory_complications30d) %>%
        mutate(Treatment = 3)
    )
phat_w_3 <- predict(xgb_v0, newdata = cov_mat_w_3)
mean(phat_w_2/phat_w_1)
mean(phat_w_2/phat_w_3)
mean(phat_w_1/phat_w_3)
```

3.7 Propensity score weighting using BART

```
library(WeightIt)
weightit_bart <-
  weightit(
    treatment ~ age + gender + married + race + income + chalson_
       comorbidity_score +
      year_of_diagnosis + cancer_stage + tumor_size_nm + histology +
          tumor_site +
      pet_scan + chest_ct + mediastinoscopy,
    data = data %>% janitor::clean_names() %>% as.data.frame(),
    method = "bart",
    estimand = "ATE"
  )
```

3.8 Propensity score weighting using RF

```
library(WeightIt)
weightit_rf <-
  weightit(
    treatment ~ age+ gender + married + race + income + chalson_
       comorbidity_score +year_of_diagnosis + cancer_stage + tumor_
       size_nm + histology + tumor_site+ pet_scan + chest_ct +
       mediastinoscopy,
    data = data %>% janitor::clean_names() %>% as.data.frame() %>%
       mutate(treatment = as.factor(treatment)),
    method = "SuperLearner",
    SL.library = c("SL.randomForest"),
    estimand = "ATE"
  )
```

3.9 Propensity score weighting using XGBoost

```
library(WeightIt)
weightit_gbm <-
  weightit(
    treatment ~ age + gender + married + race + income + chalson_
       comorbidity_score +
      year_of_diagnosis + cancer_stage + tumor_size_nm + histology +
          tumor_site +
      pet_scan + chest_ct + mediastinoscopy,
    data = data %>% janitor::clean_names() %>% as.data.frame(),
```

```r
    method = "gbm",
    estimand = "ATE"
  )
```

3.10 Missing data

```r
library(missForest)
dat_mod <- read_csv("data/dat_mets_60cov_032221.csv") %>%
  select(-X1) %>%
  mutate_if(is.character, as.factor) %>%
  as.data.frame()
dat_modmissForest <- missForest(dat_mod)
```
